# Is now the time for a Rubiscuit or Ruburger? Increased interest in Rubisco as a food protein

**DOI:** 10.1093/jxb/erac414

**Published:** 2022-10-19

**Authors:** F Grant Pearce, Joel E Brunke

**Affiliations:** Biomolecular Interactions Centre and School of Biological Sciences, University of Canterbury, Christchurch, New Zealand; Biomolecular Interactions Centre and School of Biological Sciences, University of Canterbury, Christchurch, New Zealand; Western Sydney University, Australia

**Keywords:** Food protein, green protein, leaf protein, nutritional value, processing, Rubisco

## Abstract

Much of the research on Rubisco aims at increasing crop yields, with the ultimate aim of increasing plant production to feed an increasing global population. However, since the identification of Rubisco as the most abundant protein in leaf material, it has also been touted as a direct source of dietary protein. The nutritional and functional properties of Rubisco are on a par with those of many animal proteins, and are superior to those of many other plant proteins. Purified Rubisco isolates are easily digestible, nutritionally complete, and have excellent foaming, gelling, and emulsifying properties. Despite this potential, challenges in efficiently extracting and separating Rubisco have limited its use as a global foodstuff. Leaves are lower in protein than seeds, requiring large amounts of biomass to be processed. This material normally needs to be processed quickly to avoid degradation of the final product. Extraction of Rubisco from the plant material requires breaking down the cell walls and rupturing the chloroplast. In order to obtain high-quality protein, Rubisco needs to be separated from chlorophyll, and then concentrated for final use. However, with increased consumer demand for plant protein, there is increased interest in the potential of leaf protein, and many commercial plants are now being established aimed at producing Rubisco as a food protein, with over US$60 million of funding invested in the past 5 years. Is now the time for increased use of Rubisco in food production as a nitrogen source, rather than just providing a carbon source?

## Teaser

With increased interest in Rubisco as a food protein, the review covers how research into the role of Rubisco in photosynthesis compares with research into the extraction and use of Rubisco in food systems.

## Introduction

In an interview with the New Yorker magazine in 2019, Pat Brown, founder of Impossible Foods said ‘for a year, our prototype burgers used Rubisco, and it worked functionally better than any other protein, making a juicy burger’ (New Yorker Magazine, 2019). A patent filed by Impossible Foods in 2015 (US 10,172,380) describes the use of Rubisco as a binding agent, and an example of Rubisco isolation and purification. The main issue cited for not using it in the current product was that no one produces Rubisco at scale.

As the enzyme that fixes CO_2_ from the atmosphere into organic carbon, Rubisco is a key target for studies aimed at increasing crop yield through improved photosynthetic carbon assimilation. With a relatively slow catalytic rate and inefficiency due to the competing oxygenase reaction, it has been calculated that even marginal gains in Rubisco efficiency would translate into significant gains in crop yield (Parry *et al.*, 2011). While these studies are ongoing, engineering a Rubisco enzyme with increased activity and/or higher substrate specificity has remained elusive.

Extraction of protein from leaf material had been studied even before the identification of Rubisco as the main component. In 1773, Hilaire Rouelle published work describing the extraction of a compound from leaves that had similar properties to meat. Work on leaf protein continued through the 1920s, before increased efforts during the Second World War (Pirie, 1966). Around this time, Sam Wildman carried out work on extracting soluble protein from leaf material. Initially, a class of protein was identified that formed a precipitate in the presence of 35% ammonium sulfate, termed ‘Fraction 1’. Later work identified that this fraction was homogeneous, leading to its labelling as ‘Fraction 1 protein’, before the identification of Fraction 1 protein as the carboxylation enzyme in photosynthesis in 1957. The abbreviation Rubisco was suggested by David Eisenberg in 1979, as a reference to Nabisco (the National Biscuit Company), alluding to the interests of Sam Wildman in promoting Fraction 1 protein as food (Wildman, 1992, 2002).

Many countries are experiencing growing consumer demand for plant-based proteins, due to perceived health benefits and increased concerns about animal welfare and environmental costs of animal-based proteins. Epidemiological research links cancer, cardiovascular disease, and type II diabetes mellitus with the high consumption of meat, while a plant-based diet that is high in fibre is associated with decreased risk of colorectal cancer, cardiovascular disease, type II diabetes mellitus, and overall mortality. Consumption of meat is also associated with increased greenhouse emissions and reduced ground water quality due to effluent discharge.

Crops are highly efficient at using photosynthesis to produce edible material from limited resources. In contrast, <20% of protein from plant feed crops is converted into animal protein (Pimentel and Pimentel, 2003), with the remainder being excreted, often resulting in increased nitrogen levels in waterways. Animal protein production also requires high fossil energy inputs (25 kJ of fossil energy per kJ of animal protein compared with 2.2 kJ of fossil energy per kJ of plant protein) and water resources, with up to 100 times more water being required (Pimentel and Pimentel, 2003).

Plant-based products often aim to replace meat with alternative ingredients that have similar appearance, taste, and cooking methods. Traditional meat alternatives (tofu, tempeh, and falafel) do not necessarily replicate the taste and appearance of meat directly yet are often used as substitutes during cooking. Similarly, whole foods such as jackfruit or banana blossom have a similar appearance and texture to meat. More recently, plant-based meat products aim to more accurately replicate the taste, texture, and appearance of meat. Inclusion of plant-based leghaemoglobin allows the taste and colour of meat to be more accurately replicated. Rather than wholefood ingredients, these products often use processed plant protein isolates. Despite growing consumer demand, plant-based proteins often lack the functionality (solubility, ability to form gels and foams) of animal-based proteins.

Several reviews describing the use of Rubisco as a food protein have been published over the years (Pirie, 1966; Barbeau and Kinsella, 1988; Di Stefano *et al.*, 2018; Møller *et al.*, 2021). This review aims to bridge the knowledge gained from Rubisco research focused on its role in photosynthesis with current studies of Rubisco as a food protein.

## Protein in food

Animals need protein in their diet to provide the amino acids needed for maintenance of the body. Globally, 40% of dietary protein is derived from animal sources, varying from 60% in high-income countries to ~30% in low- and middle-income countries. In 2019, global protein intake was 81.7 g of protein per capita per day, with plant-based protein accounting for 32.1 g of protein per capita per day (FAOSTAT, 2022). Of the plants, cereal crops (e.g. rice, wheat, and corn) provide the largest protein source (providing more protein than meat in Africa, Central America, Asia, and Europe), with the remainder coming from pulses (e.g. soybeans, peas, and lentils) (Poutanen *et al.*, 2022). With nearly all plant protein being derived from seeds, very little dietary protein is currently obtained from leaves.

Protein intake is often limited by the digestibility and nutritional content of the food, with animal proteins being considered of higher quality than plant proteins. The Digestible Indispensable Amino Acid Score (DIAAS) evaluates the quality of protein based on the amino acid content and the ability of the body to break down and absorb the protein in relation to the body’s requirements. Food protein digestibility depends on the protein structure, and lower accessibility due to the tough plant cell walls as well as the presence of anti-nutritional factors such as polyphenols, phytates, saponin, protease inhibitors, etc. (Livingston *et al.*, 1979; Samtiya *et al.*, 2020). Animal proteins characteristically have a high digestibility (>95% of protein taken up) compared with plant proteins (75–80%) (Sá *et al.*, 2020). Nine amino acids are unable to be synthesized by humans, making them essential in the diet. While animal proteins provide nearly all of the amino acids required by humans, lysine, methionine, histidine, and isoleucine are only found in low levels in plant ingredients (Dimina *et al.*, 2022). Current research aims to blend plant proteins from different sources to mimic the amino acid profile of animal proteins (Dimina *et al.*, 2022).

In food systems, proteins often provide functional qualities in addition to the nutritional component (Martins *et al.*, 2018). Several peptides are directly responsible for taste, including Kokumi and umami peptides. At the molecular scale, the surfaces of proteins can bind small molecules that may impart flavours, colours, or aromas. At the mesoscale, the long polymeric nature of proteins means that they can produce structures in foods such as fibres, gels (solids surrounded by liquid), or foams (gas dispersed in liquid), or act as emulsifiers to assist in the mixing of oil and water. The solubility of protein in solution is also an important aspect, with protein precipitation causing cloudiness or unfavourable mouth feel.

Plant proteins are generally considered to have less favourable functional properties than animal proteins such as whey or egg white, which naturally have excellent foaming, emulsifying, and gelling properties. The majority of plant proteins currently used in the food industry are derived from seeds rather than leaves. These include oilseeds (soybean and canola), pulses (chickpea and pea), and cereals (wheat, corn, and barley). Most proteins in seeds are storage proteins, including prolamins, albumins, glutenins, and globulins, that are characteristically insoluble. The method by which plant proteins are extracted from seeds involves alkaline extraction and precipitation, causing extensive protein denaturation and aggregation, further lowering the functional aspects of plant proteins. Products such as pea protein are often used as ingredients for meat alternatives; however, these products are generally associated with a distinct off-flavour described as ‘beany’ or ‘vegetal’ caused by aldehyde molecules associated with the pea protein (Trindler *et al.*, 2022). One new source of plant protein that is being explored is patatin (also known as tuberin) derived from potatoes. This protein also appears to be a promising candidate as a plant-based protein alternative for food production (Fu *et al.*, 2020).

Food proteins can also elicit an immune response, resulting in a food allergy. Proteins derived from plants are often found to be associated with food allergies. These include oleosin from peanuts, gluten from wheat, or non-specific lipid transfer proteins (ns-LTPs) from seeds and fruit (Maruyama, 2021). Increased incorporation of plant protein into processed foods makes it important that product labelling and manufacturing control measures are in place (Taylor, 2021).

## Potential of Rubisco as a food protein

The potential of Rubisco as a food protein lies in having a good nutritional composition, with few limiting amino acids and good digestibility, combined with low allergenicity and excellent functional properties. Many of the Rubisco isolates are colourless and odourless, with few vegetal tastes associated with them and excellent functional properties. It has been reported that Rubisco solutions can form foams as stable as those from egg white, as well as acting as emulsifiers or forming gels, highlighting the potential of Rubisco isolates as a functional food ingredient (Martin *et al.*, 2019).

### Nutritional profile

One of the limitations of plant proteins is that they are often limiting in some of the essential amino acids compared with animal proteins. In particular, cereals have low levels of lysine and methionine, while legumes have low levels of methionine (Ufaz and Galili, 2008). Part of this is a consequence of the fact that seed proteins are stable storage proteins, while leaf proteins consist of enzymes. Rubisco meets the FAO (Food and Agriculture Organization) requirements for an essential amino acid profile, being comparable with casein and other animal proteins (Hood *et al.*, 1981). Essential amino acid contents have been measured from multiple sources and adapted for comparison in Table 1 (FAO, 2011; Kalman, 2014; Wu *et al.*, 2016; Attia *et al.*, 2020; Sowersby *et al.*, 2021). The highly conserved nature of the Rubisco large subunit (Liu *et al.*, 2017) means that Rubisco will have a similar nutritional profile, independent of the plants used.

**Table 1.** Essential amino acid content as a function of total protein content from different sources compared with required DIAAS values for ages >3 years as recorded in mg g^–1^.

EAA	Rubisco (*Medicago sativa*)	Soy isolate	Whey isolate	Egg white isolate^a^	Beef^*b*^	DIAAS
Histidine	38.7	26.0	13.0	21.0	21.8	16.0
Isoleucine	48.6	48.0	56.0	55.0	27.5	30.0
Leucine	93.0	77.0	103.0	82.0	44.2	61.0
Lysine	65.3	60.0	97.0	74.4	47.8	48.0
Methionine+Cysteine	34.0	21.6	37.8	45.5	24.2	23.0
Phenylalanine+Tyrosine	128.5	78.0	52.6	93.3	41.6	41.0
Threonine	52.7	36.0	79.0	44.0	24.3	25.0
Tryptophan	26.6	13.0	19.0	12.2	6.6	6.6
Valine	67.4	47.0	59.0	58.8	32.0	40.0

^*a*^ Adapted from Attia *et al.* (2020).

^*b*^ Adapted from Wu *et al.* (2016).

### Allergenicity

Allergenicity is an important factor in any potential food-grade product; it is most accurately determined by assessing the proportion of the general population that have an allergic response to something. *In silico* methods compare peptide sequences that would result from the protein entering the human digestive tract with known sequence of allergenic effect (Krutz *et al.*, 2019). Although Rubisco has been identified as containing peptide sequences similar to those with known allergenic effects (Yakhlef *et al.*, 2021), Rubisco has very low allergenicity (Fu *et al.*, 2002; Bowman and Selgrade, 2009; Maruyama, 2021), with only one case being officially detailed (Foti *et al.*, 2012).

### Digestibility

Animal proteins (such as those from meat or milk) are easily hydrolysed by the action of proteases (>95% digestion). In contrast, unprocessed plant products often have lower digestibility (50–80%), due to the difficulty in degrading the cell wall and the presence of anti-nutritional factors. Once the cell wall and other components have been removed, plant protein isolates tend to be more digestible (Tome, 2013). Rubisco has been reported to be quickly broken down by digestive enzymes, often being degraded within seconds (Fu *et al.*, 2002; Bowman and Selgrade, 2009). A large group of contaminants are the phenolic molecules, which bind to Rubisco and increase the protein’s resistance to degradation, and therefore its nutritional value is reduced (Pedone *et al.*, 1995; Alonso *et al.*, 2000). Most recently, intervention trials have been carried out in which human subjects consumed protein concentrate derived from *Lemna* (duckweed) in order to better understand how leaf protein might be digested as part of the human diet (Mes *et al.*, 2022). This study observed no negative effects of consuming leaf protein concentrate, with less effect on glucose or insulin levels compared with whey protein concentrate, but it also showed lower uptake of amino acids compared with whey protein.

### Bioactive peptides

Upon proteolytic cleavage during the digestion process, Rubisco can form bioactive peptides, which are short sequences of amino acids that function by interacting with other proteins in the body. These peptides can have both positive and negative effects on health. Some of the peptides derived by Rubisco have been shown to have anti-bacterial (Kobbi *et al.*, 2015) or anti-hypertensive activity (Cao *et al.*, 2021; Ma *et al.*, 2021). Interestingly, two peptides derived from the Rubisco large subunit, Rubiscoin-5 (YPLDL) and Rubiscolin-6 (YPLDLF), were shown to bind to opioid receptors. Oral administration of Rubiscolin-6 to mice enhanced memory consolidation, but at doses much higher than could be achieved from dietary intake of Rubisco (Yang *et al.*, 2003). Similar studies have also shown a role for Rubiscolin-6 in reducing anxiety (Hirata *et al.*, 2007) or stimulating food intake (Kaneko *et al.*, 2012; Miyazaki *et al.*, 2014) when orally administered to mice. A recent review covers the potential for Rubisco-based bioactive peptides in more detail (Udenigwe *et al.*, 2017).

### Gelling

Rubisco can be used to form gels when combined with aqueous-based solutions; these gels are known as particulate non-transparent gels. Gels are formed when liquid is trapped in large polymer arrays (Aguilera and Stanley, 1999); these are also known as hydrocolloids or hydrogels. The effect of pH on Rubisco gels is a scale of rigidity where lower pH leads to a more brittle gel (Libouga *et al.*, 1996; Di Stefano *et al.*, 2018). The interprotein interaction capacity of a protein will determine the gelling ability of that protein. Critical gelation of Rubisco is typically achieved at lower concentrations than whey and soy isolate gels (Martin *et al.*, 2014, 2019). Heat set gels formed from spinach Rubisco were comparable with those from whey and egg white protein, with a low gelling concentration and a high gel strength, making them useful as a functional food ingredient (Martin *et al.*, 2014). Similarly, Rubisco isolates from sugar beet leaves or duckweed formed self-supporting gels that were similar to those of egg white and soy (Martin *et al.*, 2019; Nieuwland *et al.*, 2021). Gels from mulberry leaf protein concentrate (Sun *et al.*, 2015) have also been reported.

In plants, transglutaminase enzymes have been found to play a role in regulating photosynthesis (Zhong *et al.*, 2019), with the large subunit of lucerne Rubisco acting as a major substrate for transglutaminase (Margosiak *et al.*, 1990; Serafini-Fracassini and Del Duca, 2008). Commercial food processing sometimes uses the transglutaminase enzyme to form cross-links between proteins in processed meat and fish products. This observation raises the possibility that transglutaminase could be used to produce stronger Rubisco-based gels in the future.

### Emulsification

Proteins such as Rubisco have surface-active functionality, enabling them to bundle around oil in a hydrophilic environment (O/W emulsion), thereby facilitating oil suspension in water also known as emulsification (Zayas, 1997). This functionality of soluble Rubisco increases the potential uses for the enzyme as a natural creaming agent, therefore opening up possibilities as an alternative component in various foods (Hailing and Walstra, 1981). Uses as an emulsifying agent in food are primarily to aid in the shelf-life of products by separating fats from hydrophilic material (Wang *et al.*, 2020). Rubisco extracted from sugar beet leaves had similar emulsifying properties to those of whey protein isolates (Martin *et al.*, 2019). Leaf protein concentrates from mulberry (Sun *et al.*, 2015), dried alfalfa leaves (Hojilla-Evangelista *et al.*, 2017), eggplant (Famuwagun *et al.*, 2020), vegetable byproducts (Sedlar *et al.*, 2021), radish (Kaur and Bhatia, 2022), and duckweed (Tan *et al.*, 2022) have also all been shown to act as good emulsifiers. Like many of the functional characteristics of Rubisco, their specific nature is dependent on the extraction techniques used (Lamsal *et al.*, 2007).

### Foaming

Foam is formed when surface-active compounds surround a gas phase generally as small bubbles (Zayas, 1997; Narsimhan and Xiang, 2018). The required protein characteristics include appropriate unfolding speeds and general layer coherence leading to steric stability, and the ability to absorb to a biphasic interface (Lamsal *et al.*, 2007; Hojilla-Evangelista *et al.*, 2017). The foaming capacity of Rubisco will allow its use in various food types and can be tailored by adjusting the pH of the system where the strongest foams can be obtained at pH 4.5, the pI of Rubisco (Barbeau and Kinsella, 1988). The foaming capacity of Rubisco protein isolate derived from sugar beet leaves was substantially higher than that seen for whey and soy isolate where higher concentrations resulted in enhanced foaming capacity (Martin *et al.*, 2019). Above pH 6, the foams were unstable given the increased repulsion between proteins (Hojilla-Evangelista *et al.*, 2017). Rubisco isolates from alfalfa (Nissen *et al.*, 2021), vegetable byproducts (Famuwagun *et al.*, 2020; Sedlar *et al.*, 2021), and mulberry (Sun *et al.*, 2015) have all been shown to have excellent foaming capacity.

## Source of biomass

Rubisco has often been described as the most abundant protein on Earth (Ellis, 1979), and a recent updated and rigorous analysis concluded that there is ~0.7 Gt of Rubisco on the planet (Bar-On and Milo, 2019). Based on work from Onoda *et al.* (2017), Rubisco is estimated as making up ~3% of the total dry mass of leaves. The moisture content of leaves varies between species (Yeoh and Wee, 1994), but if the moisture content of the leaf material is ~80%, the proportion of Rubisco represents ~0.6% of the fresh leaf mass. If this is the case, then ~6 kg of Rubisco could be purified from 1 t of fresh leaf material.

While purification of Rubisco in the lab often focused on model organisms such as tobacco, wheat, or Arabidopsis, extraction of Rubisco as a food protein requires large amounts of biomass. Several studies have focused on current crops, such as alfalfa, which could be grown purely to produce leaf protein, or *Moringa* (Benhammouche *et al.*, 2021), a fast growing and drought-tolerant tree species. Others have investigated the extraction of protein out of the byproducts from other crops (Sedlar *et al.*, 2021), including sugar beet, cauliflower (Xu *et al.*, 2017), kale, broccoli (Prade *et al.*, 2021), radish (Kaur and Bhatia, 2022), chicory (Ducrocq *et al.*, 2022), mulberry (Sun *et al.*, 2015), Jerusalem artichokes (Kaszás *et al.*, 2022), or even invasive plant species such as gorse or broom (Iyer *et al.*, 2021). Aquatic plants, such as duckweed (*Lemna*), are also being studied (Nieuwland *et al.*, 2021).

This calculated value of Rubisco content seems to agree well with different purification methods. Laboratory purification of Rubisco reported a yield of 75 mg of Rubisco from 30 g of Arabidopsis florets (0.25% of fresh leaf mass) (Carmo-Silva *et al.*, 2011). At the industrial scale, purification of Rubisco from sugar beet leaves reported a yield of 0.67% (Tamayo Tenorio *et al.*, 2016), while a purification method from lucerne yielded 0.3% (Edwards *et al.*, 1975). Extraction of isolated Rubisco from spinach yielded 0.3% (Martin et al., 2018), and trials of different extraction methods using duckweed also reported a yield of 0.3% (Nieuwland *et al.*, 2021). Some trials have reported higher yields (3.9% for Jerusalem artichoke and 3.2% for lucerne); however, these samples were not as highly purified as other methods, and are likely to include other components as well (Kaszás *et al.*, 2022).

## Extraction and purification of Rubisco

Protein extraction from plant seeds is often easier than from leaves because seeds have a higher protein content per fresh weight (typically ~30% of fresh weight for soy beans or peas). Given that protein represents a relatively small proportion of the leaf mass (~3.8 g of protein per 100 g for dicots and ~1.7 g of protein per 100 g for monocots; Yeoh and Wee, 1994), partly due to the higher water content, large amounts of leaf material need to be processed in order to obtain sufficient protein. This also has implications for the transport and storage of material.

Protein extraction from seeds also has the advantage that seeds are often left in the field until convenient to harvest, and can be stored in grain bins until processing. In contrast, leaf material needs to be processed as soon as possible after harvest to prevent spoilage due to enzymatic activity and microbial action. Pilot plants for the processing of lucerne describe how plant material was processed within 1–2 h of harvest (Edwards *et al.*, 1975; Fiorentini and Galoppini, 1981). Alternatively, leaves can be frozen or dried before processing. While several small-scale studies freeze plant material for storage before processing (Nynäs *et al.*, 2021), and freezing does not appear to be detrimental to subsequent processing, large-scale freezing of plant material has a high energy cost (Tamayo Tenorio *et al.*, 2017). Similarly, drying the leaves also has a high energy cost, and extraction of protein from dried leaves is impaired by low yields and purity (Hojilla-Evangelista *et al.*, 2017).

Extraction and purification of Rubisco are frequently carried out in the lab for physiological and functional studies (Carmo-Silva *et al.*, 2011). These studies normally use a small amount of leaf material (typically <100 g), which is frozen with liquid nitrogen and subsequently ground with a mortar and pestle or added to a blender for the extraction of Rubisco. Protease inhibitors such as phenylmethylsulfonyl flouride (PMSF) and leupeptin are added to prevent degradation of the protein, and compounds such as polyvinylpolypyrrolidone (PVPP) reduce binding of phenolic compounds. Subsequent filtration and centrifugation remove the cell walls and membranes. Initial purification of Rubisco can then be carried out using precipitation with ammonium sulfate or polyethyl glycol, with further purification using sucrose gradient centrifugation (Goldthwaite and Bogorad, 1971). Rubisco has been purified by crystallization with high yields and purity from the tobacco plant (*Nicotiana tabacum*) (Johal *et al.*, 1980). Chromatographic methods for separating Rubisco from its environment such as ion exchange chromatography (Salvucci *et al.*, 1986), size exclusion chromatography, and a combination of both have been successful in obtaining highly purified Rubisco. Hydrophobic interaction chromatography was also used to purify various forms of Rubisco (Kreel and Tabita, 2007; O’Donnelly *et al.*, 2014). These methods produce a highly pure Rubisco preparation, suitable for detailed kinetic or structural studies; the natural abundance of Rubisco in leaf material provides protein yields far higher than many other plant proteins. At lab scale and for the purification of Rubisco in milligram amounts, these methods comprise the more effective approaches; however, for pilot-scale purifications, they remain inappropriate from a cost–benefit perspective.

### Challenge 1: the relatively low protein content of leaves compared with seeds

Large-scale extraction of Rubisco requires the use of different methods that can cope with large amounts of plant material, while using ingredients that are food safe. Feasibility studies have shown promising avenues requiring further research into their realistic potential for protein extraction from green leaves (Tamayo Tenorio *et al.*, 2017; Sowersby *et al.*, 2021). Evaluating the different methods available for protein extraction from green leaves is a necessary component of any commercializing enterprise. Investigations into possible approaches show a positive influence in terms of energy use and environmental impact when the number of products from a single process is maximized and when steps are increased to enhance product purity (Corona *et al.*, 2018).

**Fig. 1.**
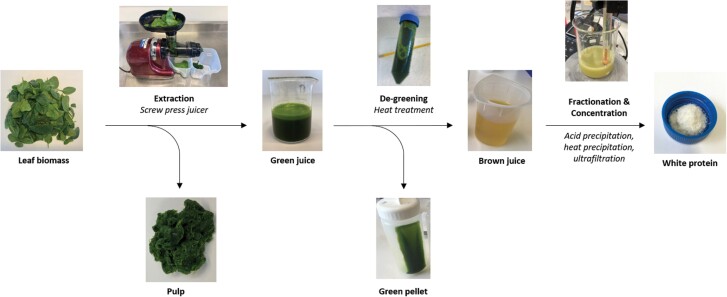
Overview of the extraction of protein from leaf biomass.

Unlike protein extraction out of animal-derived products (e.g. milk), the protein in leaves is enclosed inside the tough cell wall, which is strengthened with cellulose. Macerating the leaf material is most frequently carried out using a twin-screw press, which has two overlapping screws that squeeze the leaf material against a screen, allowing the juice to be collected (Fig. 1). Other methods of extraction include sugar cane rolls, hammer mills, or shredders, but the use of twin-screw presses has been the main method described for plant material such as sugar beet leaves, lucerne, or other crop plants (Tamayo Tenerio *et al*., 2017; Edwards *et al.*, 1975; Nynäs *et al.*, 2021). During the extraction process, different compounds are sometimes included to improve the yield of product, as described for the extraction of chlorophyll from spinach (Özkan and Bilek, 2015). Given that the Rubisco is located inside the chloroplasts, if can be difficult to completely break open the cell and chloroplast membranes to release the protein. Inclusion of cellulase, hemicellulase, or pectinase enzymes during processing has been suggested to increase protein yields by breaking down the cell wall (Akyüz and Ersus, 2021).

As soon as the leaf material has been disrupted, browning can occur by the action of polyphenol oxidase, which catalyses the formation of diphenols and quinones. The quinone compounds can react with themselves to form brown pigments, or with proteins. The presence of these brown compounds reduces the consumer acceptance of the final product while reducing the functional and nutritional characteristics of the protein. Although PVPP is commonly added to reduce browning during laboratory-scale purifications (Carmo-Silva *et al.*, 2011), this is not suitable at a large scale. Compounds such as ascorbic acid or metabisulfite are antioxidants that inhibit the activity of polyphenol oxidase, reducing browning.

### Challenge 2: removal of chlorophyll

One of the biggest challenges in producing food protein is the extraction and fractionation of Rubisco from other components of the leaf. In particular, green chlorophyll needs to be removed in order to produce a colourless compound, along with small molecules that are associated with bitter or ‘vegetal’ tastes, and the material needs to be concentrated into a useful form (Fig. 1). Several biorefineries have been developed that simply precipitate all proteins from the juice, producing a leaf protein concentrate that is suitable for animal feed. From a consumer perspective, the green colour of the chlorophyll, along with undesirable taste and small components, make the product less attractive. Green juice is composed of soluble (mainly Rubisco) and insoluble protein along with large cell debris such as cell walls and fractured organelles. At lab scale, high centrifugation speeds are feasible; however, alternative approaches are required when upscaling the extraction process.

Maintaining native-structure Rubisco throughout the extraction process is often crucial for the final product; in this case, the methods used must ensure Rubisco is not denatured. One simple process is to heat the juice solution to 50–55 °C for 20–30 min, which causes a majority of chlorophyll and associated proteins (‘green protein’) to aggregate (Tamayo Tenorio *et al.*, 2016; Nynäs *et al.*, 2021). This temperature is below that necessary to denature Rubisco, and the precipitated compounds can be removed by centrifugation or decanting.

### Challenge 3: purification of high quality protein

In some instances, initial removal of the green proteins is considered sufficient, and the resulting supernatant can be freeze-dried to produce a protein isolate (Tamayo Tenorio *et al.*, 2016). However, other techniques are often used to both concentrate the ‘white protein’ from the supernatant and separate it from other small molecules that may be associated with taste, colour, aroma, or functionality (Fig. 1). pH precipitation is often used during large-scale Rubisco extractions (Yang *et al.*, 2004). In this case, the pH is lowered to 4.5, close to the isoelectric point of Rubisco. This causes the Rubisco to precipitate for recovery by centrifugation (Nynäs *et al.*, 2021). Heating to 80 °C can also be used to purify the white fraction containing Rubisco. This method leads to the denaturation and gelation of Rubisco upon cooling (Zayas, 1997). Treatment methods are generally tailored to reflect desired characteristics in the final product such as solubility; ensuring the lowest stress on the protein during extraction and purification improves the soluble yield.

During food processing, membrane filtration is frequently used to purify the final product by concentrating the solution and removing unwanted components. Microfiltration used a membrane with a larger pore size that excludes bacteria, whole cells, or chloroplasts. Many lab researchers will be familiar with centrifugation concentration devices, in which ultrafiltration is used to separate particles in solution. By using a membrane with a cut-off that is lower than that of the protein, water and small molecules can be removed from the sample. Diafiltration, designed to retain the protein fraction, has been employed in sequence with the previously described filtration method to effectively screen against small molecular weight contaminants (Knuckles *et al.*, 1975; Zhang *et al.*, 2015). An example of this has been described for duckweed protein concentrate, in which the solution was first passed through a 0.45 µm membrane (microfiltration) to remove microbes and green material, followed by ultrafiltration with a 100 kDa membrane (Nieuwland *et al.*, 2021).

Washing steps are often used to increase product quality by removing contaminants such as phenols, which interact with the protein fraction and are purified with it (Wang and Xiong, 2019). Besides the accompanying contaminants in the final product, membrane fouling reduces the effectiveness of using filtration techniques to purify Rubisco. Protein can become associated with the concentrating layer of non-permeateing material, thereby reducing obtainable yields (Kumar and Ismail, 2015). Some approaches use continuous flow membrane systems where the fouling layer is constantly washed away and the permeate volume is replaced in the feed by buffers; this can increase protein flux while mitigating concentration effects. Such methods can suffer when impurities such as phenols, lipids, and colouring agents (carotenoids) remain with the protein fraction; this is only a concern when permeating the protein fraction (Fernández *et al.*, 2007; Zhang *et al.*, 2015). Increases in temperature improve membrane flux while reducing membrane specificity (Zhang *et al.*, 2017). Adjusting parameters such as pH, temperature, and salinity during the extraction process affects changes in the soluble yields and functional properties of protein products (Lamsal *et al.*, 2007; Nissen *et al.*, 2021).

## Conclusions

Using leaf biomass as a source of food protein has challenges associated with the naturally low protein content due to a high water content, necessitating large amounts of material to be processed, and providing challenges for transportation. The biomass also needs to be processed quickly, and is often seasonal. Extraction of high-quality protein requires removal of the chlorophyll content, while maintaining the native structure of Rubisco.

Plant-based protein currently earns a revenue of ~US$8–14 kg^–1^ (Prade *et al.*, 2021). An analysis of broccoli and kale leaves concluded that full fractionation of the biomass is currently not economically feasible due to the low yields of white protein concentrate, but that increased yields or development of added value co-products would contribute to economic viability (Prade *et al.*, 2021). A study of the potential for New Zealand to produce leaf-based plant proteins found the break-even point would be ~1000 ha of pasture crop, which would produce ~4200 t of protein (Sowersby *et al.*, 2021). It is estimated that the manufacturing facility required to process the material would cost approximately US$64 million (Sowersby *et al.* 2021).

While the exact model used for commercial operations is unknown, several concepts have been discussed for a possible supply chain. In order to reduce transport load, extraction of juice could be carried out on farms using mobile units before chilled juice is transported to a central processing factory (Tamayo Tenorio *et al.*, 2017). Maximizing the economic benefits requires an integrated process that needs to use as many of the co-products as possible. One significant co-product is the fibrous pulp produced during juicing and one of the simplest options is to return this material to the land to fertilize the soil (Tamayo Tenorio *et al.*, 2017). Given that there is still a significant protein content in the pulp, it can also be used as animal fodder. A potential higher value option is to use this material, which has a high cellulose content, as a substrate for bio-ethanol production.

Despite these challenges, there are currently several groups working towards developing commercial products based on leaf proteins. An EU-funded project from 2016 to 2021 (CORDIS_720728, €5.5 million) aimed to develop a demonstration plant capable of processing 1.5 t of sugar beet leaves per hour to produce 28 kg of Rubisco. In 2021, Plantible Foods (based in San Diego) announced that it had raised US$27million to date with the aim of recovering protein from duckweed (Vegconomist, 2021). Leaft foods, based in New Zealand, announced US$15 million in additional funding in 2022, following US$13 million in 2021 (Leaft Foods, 2022). This company aims to ‘capture the most plentiful protein on the planet, Rubisco from green leaves’. In Sweden, the Plant Protein Factory will use green biomass to produce highly processed plant proteins (Vinnova, 2021), while Rubisco Foods opened a factory in the Netherlands in 2020 (Rubisco Foods, 2020).

**Fig. 2.**
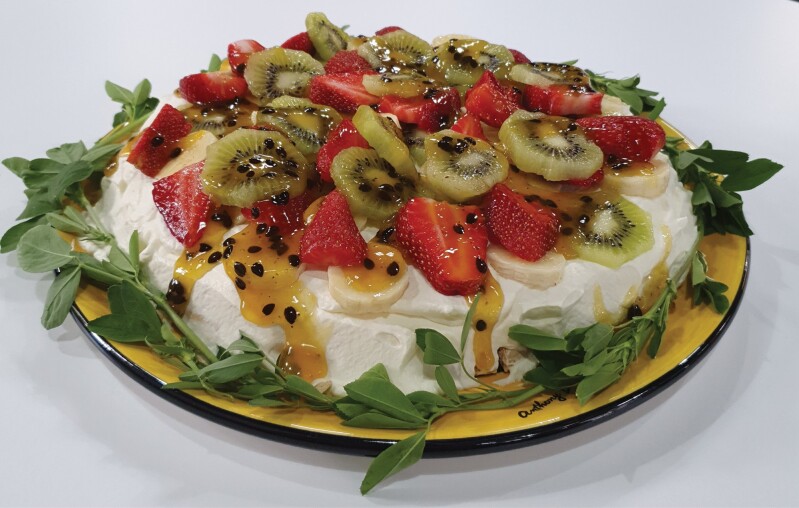
Example of a food, in this case a pavlova, made from leaf protein concentrate (image kindly supplied by Leaft Foods).

The high functionality of Rubisco means that it will most probably be incorporated into high-value foods, rather than as a bulk food additive. Highly soluble proteins that have good foaming, emulsifying, and gelling qualities can be used to make products that have high consumer appeal. A recent example showed that Rubisco protein formed cross-links with gluten when added to bread dough (Ducrocq *et al.*, 2020). Leaft Foods used leaf protein to make a pavlova, rather than eggs (Fig. 2; Stuff, 2021). With increasing demand for plant-based proteins in the diet, it may be that the time has come when commercial production of leaf proteins will result in more and more consumer proteins containing Rubisco.

## References

[CIT0001] Aguilera JM , Stanley DW. 1999. Microstructural principles of food processing and engineering. New York: Springer.

[CIT0002] Akyüz A , Ersus S. 2021. Optimization of enzyme assisted extraction of protein from the sugar beet (*Beta vulgaris* L.) leaves for alternative plant protein concentrate production. Food Chemistry 335, 127673. 32745844 10.1016/j.foodchem.2020.127673

[CIT0003] Alonso R , Aguirre A, Marzo F. 2000. Effects of extrusion and traditional processing methods on antinutrients and in vitro digestibility of protein and starch in faba and kidney beans. Food Chemistry 68, 159–165.

[CIT0004] Attia YA , Al-Harthi MA, Korish MA, Shiboob MH. 2020. Protein and amino acid content in four brands of commercial table eggs in retail markets in relation to human requirements. Animals 10, 406. 32121495 10.3390/ani10030406 PMC7142600

[CIT0005] Barbeau WE , Kinsella JE. 1988. Ribulose bisphosphate carboxylase/oxygenase (rubisco) from green leaves—potential as a food protein. Food Reviews International 4, 93–127.

[CIT0006] Bar-On YM , Milo R. 2019. The global mass and average rate of rubisco. Proceedings of the National Academy of Sciences, USA 116, 4738–4743. 10.1073/pnas.1816654116 PMC6410859 30782794

[CIT0007] Benhammouche T , Melo A, Martins Z, Faria MA, Pinho SCM, Ferreira IMLPVO, Zaidi F. 2021. Nutritional quality of protein concentrates from *Moringa oleifera* leaves and in vitro digestibility. Food Chemistry 348, 128858. 33508602 10.1016/j.foodchem.2020.128858

[CIT0008] Bowman CC , Selgrade MK. 2009. Utility of rodent models for evaluating protein allergenicity. Regulatory Toxicology and Pharmacology 54, S58–S61. 18957311 10.1016/j.yrtph.2008.10.002

[CIT0009] Cao X , Yang J, Ma H, Guo P, Cai Y, Xu H, Ding G, Gao D. 2021. Angiotensin I converting enzyme (ACE) inhibitory peptides derived from alfalfa (*Medicago sativa* L.) leaf protein and its membrane fractions. Journal of Food Processing and Preservation 45, e15834.

[CIT0010] Carmo-Silva AE , Barta C, Salvucci ME. 2011. Isolation of ribulose-1,5-bisphosphate carboxylase/oxygenase from leaves. Methods in Molecular Biology 684, 339–347. 20960141 10.1007/978-1-60761-925-3_26

[CIT0011] Corona A , Ambye-Jensen M, Vega GC, Hauschild MZ, Birkved M. 2018. Techno-environmental assessment of the green biorefinery concept: combining process simulation and life cycle assessment at an early design stage. Science of the Total Environment 635, 100–111. 29660714 10.1016/j.scitotenv.2018.03.357

[CIT0012] Di Stefano E , Agyei D, Njoku EN, Udenigwe CC. 2018. Plant RuBisCo: an underutilized protein for food applications. Journal of the American Oil Chemists’ Society 95, 1063–1074.

[CIT0013] Dimina L , Rémond D, Huneau J-F, Mariotti F. 2022. Combining plant proteins to achieve amino acid profiles adapted to various nutritional objectives—an exploratory analysis using linear programming. Frontiers in Nutrition 8, 809685. 35187024 10.3389/fnut.2021.809685 PMC8850771

[CIT0014] Ducrocq M , Boire A, Anton M, Micard V, Morel MH. 2020. Rubisco: a promising plant protein to enrich wheat-based food without impairing dough viscoelasticity and protein polymerisation. Food Hydrocolloids 109, 106101.

[CIT0015] Ducrocq M , Morel MH, Anton M, Micard V, Guyot S, Beaumal V, Solé-Jamault V, Boire A. 2022. Biochemical and physical–chemical characterisation of leaf proteins extracted from *Cichorium endivia* leaves. Food Chemistry 381, 132254. 35124496 10.1016/j.foodchem.2022.132254

[CIT0016] Edwards RH , Miller RE, de Fremery D., Knuckles BE, Bickoff EM, Kohler GO. 1975. Pilot plant production of an edible white fraction leaf protein concentrate from alfalfa. Journal of Agricultural and Food Chemistry 23, 620–626.

[CIT0017] Ellis RJ. 1979. The most abundant protein in the world. Trends in Biochemical Sciences 11, 241–244.

[CIT0018] Famuwagun AA , Alashi AM, Gbadamosi SO, Taiwo KA, Oyedele DJ, Adebooye OC, Aluko RE. 2020. Comparative study of the structural and functional properties of protein isolates prepared from edible vegetable leaves. International Journal of Food Properties 23, 955–970.

[CIT0019] FAO. 2011. Dietary protein quality evaluation in human nutrition. FAO Food and Nutrition Paper. Rome: Food and Agriculature Organization.

[CIT0020] FAOSTAT. 2022. Food and Agriculture Organization of the United Nations. https://www.fao.org/faostat/en/#home

[CIT0021] Fernández SS , Menéndez C, Mucciarelli S, Padilla AP. 2007. Saltbush (*Atriplex lampa*) leaf protein concentrate by ultrafiltration for use in balanced animal feed formulations. Journal of the Science of Food and Agriculture 87, 1850–1857.

[CIT0022] Fiorentini R , Galoppini C. 1981. Pilot plant production of an edible alfalfa protein concentrate. Journal of Food Science 46, 1514–1517.

[CIT0023] Foti C , Damiani E, Zambonin CG, Cassano N, Nettis E, Ferrannini A, Calvano CD, Aresta A, Romita P, Aloia AM. 2012. Urticaria and angioedema to rubisco allergen in spinach and tomato. Annals of Allergy, Asthma & Immunology 108, 60–61. 10.1016/j.anai.2011.09.011 22192968

[CIT0024] Fu TJ , Abbott UR, Hatzos C. 2002. Digestibility of food allergens and nonallergenic proteins in simulated gastric fluid and simulated intestinal fluid—a comparative study. Journal of Agricultural and Food Chemistry 50, 7154–7160. 12428975 10.1021/jf020599h

[CIT0025] Fu Y , Liu W-N, Soladoye OP. 2020. Towards potato protein utilisation: insights into separation, functionality and bioactivity of patatin. International Journal of Food Science & Technology 55, 2314–2322.

[CIT0026] Goldthwaite JJ , Bogorad L. 1971. A one-step method for the isolation and determination of leaf ribulose-1,5-diphosphate carboxylase. Analytical Biochemistry 41, 57–66. 5578554 10.1016/0003-2697(71)90191-6

[CIT0027] Hailing PJ , Walstra P. 1981. Protein-stabilized foams and emulsions. Critical Reviews in Food Science & Nutrition 15, 155–203. 7023848 10.1080/10408398109527315

[CIT0028] Hirata H , Sonoda S, Agui S, Yoshida M, Ohinata K, Yoshikawa M. 2007. Rubiscolin-6, a delta opioid peptide derived from spinach Rubisco, has anxiolytic effect via activating sigma1 and dopamine D1 receptors. Peptides 28, 1998–2003. 17766012 10.1016/j.peptides.2007.07.024

[CIT0029] Hojilla-Evangelista MP , Selling GW, Hatfield R, Digman M. 2017. Extraction, composition, and functional properties of dried alfalfa (*Medicago sativa* L.) leaf protein. Journal of the Science of Food and Agriculture 97, 882–888. 27198121 10.1002/jsfa.7810

[CIT0030] Hood L , Cheng S, Koch U, Brunner J. 1981. Alfalfa proteins: isolation and partial characterization of the major component—Fraction I protein. Journal of Food Science 46, 1843–1850.

[CIT0031] Iyer A , Bestwick CS, Duncan SH, Russell WR. 2021. Invasive plants are a valuable alternate protein source and can contribute to meeting climate change targets. Frontiers in Sustainable Food Systems 5, 575056.

[CIT0032] Johal S , Bourque DP, Smith WW, Suh SW, Eisenberg D. 1980. Crystallization and characterization of ribulose 1,5-bisphosphate carboxylase/oxygenase from eight plant species. Journal of Biological Chemistry 255, 8873–8880. 7410399

[CIT0033] Kalman DS. 2014. Amino acid composition of an organic brown rice protein concentrate and isolate compared to soy and whey concentrates and isolates. Foods 3, 394–402. 28234326 10.3390/foods3030394 PMC5302255

[CIT0034] Kaneko K , Lazarus M, Miyamoto C, et al. 2012. Orally administered rubiscolin-6, a delta opioid peptide derived from Rubisco, stimulates food intake via leptomeningeal lipocallin-type prostaglandin D synthase in mice. Molecular Nutrition and Food Research 56, 1315–1323. 22715053 10.1002/mnfr.201200155

[CIT0035] Kaszás L , Alshaal T, Kovács Z, Koroknai J, Elhawat N, Nagy E, El-Ramady H, Fári M, Domokos-Szabolcsy E. 2022. Refining high-quality leaf protein and valuable co-products from green biomass of Jerusalem artichoke (*Helianthus tuberosus* L.) for sustainable protein supply. Biomass Conversion and Biorefinery 12, 2149–2164.

[CIT0036] Kaur G , Bhatia S. 2022. Radish leaf protein concentrates: optimization of alkaline extraction for production and characterization of an alternative plant protein concentrate. Journal of Food Measurement and Characterization 16, 3166–3181.

[CIT0037] Knuckles BE , De Fremery D, Bickoff E, Kohler GO. 1975. Soluble protein from alfalfa juice by membrane filtration. Journal of Agricultural and Food Chemistry 23, 209–212. 237026 10.1021/jf60198a030

[CIT0038] Kobbi S , Balti R, Bougatef A, Le Flem G, Firdaous L, Bigan M, Chataigné G, Chaabouni S, Dhulster P, Nedjar N. 2015. Antibacterial activity of novel peptides isolated from protein hydrolysates of RuBisCO purified from green juice alfalfa. Journal of Functional Foods 18, 703–713.

[CIT0039] Kreel NE , Tabita FR. 2007. Substitutions at methionine 295 of *Archaeoglobus fulgidus* ribulose-1,5-bisphosphate carboxylase/oxygenase affect oxygen binding and CO_2_/O_2_ specificity. Journal of Biological Chemistry 282, 1341–1351. 17074752 10.1074/jbc.M609399200

[CIT0040] Krutz NL , Winget J, Ryan CA, Wimalasena R, Maurer-Stroh S, Dearman RJ, Kimber I, Gerberick GF. 2019. Proteomic and bioinformatic analyses for the identification of proteins with low allergenic potential for hazard assessment. Toxicological Sciences 170, 210–222. 30903174 10.1093/toxsci/kfz078

[CIT0041] Kumar R , Ismail A. 2015. Fouling control on microfiltration/ultrafiltration membranes: effects of morphology, hydrophilicity, and charge. Journal of Applied Polymer Science 132.

[CIT0042] Lamsal B , Koegel R, Gunasekaran S. 2007. Some physicochemical and functional properties of alfalfa soluble leaf proteins. LWT Food Science and Technology 40, 1520–1526.

[CIT0043] Leaft Foods. 2022. $15mUSD Series A accelerates mission to create new protein system https://www.leaftfoods.com/newsroom/leaft-foods-attracted-the-attention-of-international/

[CIT0044] Libouga DG , Aguié-Béghin V, Douillard R. 1996. Thermal denaturation and gelation of rubisco: effects of pH and ions. International Journal of Biological Macromolecules 19, 271–277. 9024903 10.1016/s0141-8130(96)01137-3

[CIT0045] Liu D , Ramya RCS, Mueller-Cajar O. 2017. Surveying the expanding prokaryotic Rubisco multiverse. FEMS Microbiology Letters 364, fnx156. 10.1093/femsle/fnx156 28854711

[CIT0046] Livingston AL , Knuckles BE, Edwards RH, De Fremery D, Miller RE, Kohler GO. 1979. Distribution of saponin in alfalfa protein recovery systems. Journal of Agricultural and Food Chemistry 27, 362–365. 34639 10.1021/jf60222a051

[CIT0047] Ma K , Wang Y, Wang M, Wang Z, Wang X, Ju X, He R. 2021. Antihypertensive activity of the ACE–renin inhibitory peptide derived from *Moringa oleifera* protein. Food and Function 12, 8994–9006. 34382048 10.1039/d1fo01103k

[CIT0048] Margosiak SA , Dharma A, Bruce-Carver MR, Gonzales AP, Louie D, Kuehn GD. 1990. Identification of the large subunit of ribulose 1, 5-bisphosphate carboxylase/oxygenase as a substrate for transglutaminase in *Medicago sativa* L. (alfalfa). Plant Physiology 92, 88–96. 16667270 10.1104/pp.92.1.88 PMC1062252

[CIT0049] Martin AH , Castellani O, de Jong GAH, Bovetto L, Schmitt C. 2019. Comparison of the functional properties of RuBisCO protein isolate extracted from sugar beet leaves with commercial whey protein and soy protein isolates. Journal of the Science of Food and Agriculture 99, 1568–1576. 30144065 10.1002/jsfa.9335

[CIT0050] Martin AH , Nieuwland M, de Jong GA. 2014. Characterization of heat-set gels from RuBisCO in comparison to those from other proteins. Journal of Agricultural and Food Chemistry 62, 10783–10791. 25314325 10.1021/jf502905g

[CIT0051] Martins JT , Bourbon AI, Pinheiro AC, Fasolin LH, Vicente AA. 2018. Protein-based structures for food applications: from macro to nanoscale. Frontiers in Sustainable Food Systems 2, 10.3389/fsufs.2018.00077.

[CIT0052] Maruyama N. 2021. Components of plant-derived food allergens: structure, diagnostics, and immunotherapy. Allergology International 70, 291–302. 34092500 10.1016/j.alit.2021.05.001

[CIT0053] Mes JJ , Esser D, Oosterink E, van den Dool RTM, Engel J, de Jong GAH, Wehrens R, van der Meer IM. 2022. A controlled human intervention trial to study protein quality by amino acid uptake kinetics with the novel *Lemna* protein concentrate as case study. International Journal of Food Sciences and Nutrition 73, 251–262. 34407734 10.1080/09637486.2021.1960958

[CIT0054] Miyazaki Y , Kaneko K, Iguchi S, Mizushige T, Kanamoto R, Yoshikawa M, Shimizu T, Ohinata K. 2014. Orally administered δ opioid agonist peptide rubiscolin-6 stimulates food intake in aged mice with ghrelin resistance. Molecular Nutrition and Food Research 58, 2046–2052. 25047666 10.1002/mnfr.201400100

[CIT0055] Møller AH , Hammershøj M, Dos Passos NHM, Tanambell H, Stødkilde L, Ambye-Jensen M, Danielsen M, Jensen SK, Dalsgaard TK. 2021. Biorefinery of green biomass-how to extract and evaluate high quality leaf protein for food? Journal of Agricultural and Food Chemistry 69, 14341–14357. 34845908 10.1021/acs.jafc.1c04289

[CIT0056] Narsimhan G , Xiang N. 2018. Role of proteins on formation, drainage, and stability of liquid food foams. Annual Review of Food Science and Technology 9, 45–63. 10.1146/annurev-food-030216-030009 29272186

[CIT0057] New Yorker Magazine. 2019. Can a Burger Help Solve Climate Change? https://www.newyorker.com/magazine/2019/09/30/can-a-burger-help-solve-climate-change

[CIT0058] Nieuwland M , Geerdink P, Engelen-Smit NPE, van der Meer IM, America AHP, Mes JJ, Kootstra AMJ, Henket JTMM, Mulder WJ. 2021. Isolation and gelling properties of duckweed protein concentrate. ACS Food Science and Technology 1, 908–916.

[CIT0059] Nissen SH , Schmidt JM, Gregersen S, Hammershøj M, Møller AH, Danielsen M, Stødkilde L, Nebel C, Dalsgaard TK. 2021. Increased solubility and functional properties of precipitated alfalfa protein concentrate subjected to pH shift processes. Food Hydrocolloids 119, 106874.

[CIT0060] Nynäs AL , Newson WR, Johansson E. 2021. Protein fractionation of green leaves as an underutilized food source—protein yield and the effect of process parameters. Foods 10, 2533. 34828813 10.3390/foods10112533 PMC8622718

[CIT0061] O’Donnelly K , Zhao G, Patel P, Butt MS, Mak LH, Kretschmer S, Woscholski R, Barter LMC. 2014. Isolation and kinetic characterisation of hydrophobically distinct populations of form I Rubisco. Plant Methods 10, 17. 24987448 10.1186/1746-4811-10-17 PMC4076768

[CIT0062] Onoda Y , Wright IJ, Evans JR, Hikosaka K, Kitajima K, Niinemets U, Poorter H, Tosens T, Westoby M. 2017. Physiological and structural tradeoffs underlying the leaf economics spectrum. New Phytologist 214, 1447–1463. 28295374 10.1111/nph.14496

[CIT0063] Özkan G , Bilek SE. 2015. Enzyme-assisted extraction of stabilized chlorophyll from spinach. Food Chemistry 176, 152–157. 25624218 10.1016/j.foodchem.2014.12.059

[CIT0064] Parry MA , Reynolds M, Salvucci ME, Raines C, Andralojc PJ, Zhu XG, Price GD, Condon AG, Furbank RT. 2011. Raising yield potential of wheat. II. Increasing photosynthetic capacity and efficiency. Journal of Experimental Botany 62, 453–467. 21030385 10.1093/jxb/erq304

[CIT0065] Pedone S , Selvaggini R, Fantozzi P. 1995. Leaf protein availability in food: significance of the binding of phenolic compounds to ribulose-1,5-diphosphate carboxylase. LWT Food Science and Technology 28, 625–634.

[CIT0066] Pimentel D , Pimentel M. 2003. Sustainability of meat-based and plant-based diets and the environment. American Journal of Clinical Nutrition 78, 660S–663S. 12936963 10.1093/ajcn/78.3.660S

[CIT0067] Pirie NW. 1966. Leaf protein as a human food. Science 152, 1701–1705. 5328118 10.1126/science.152.3730.1701

[CIT0068] Poutanen KS , Kårlund AO, Gómez-Gallego C, et al. 2022. Grains—a major source of sustainable protein for health. Nutrition Reviews 80, 1648–1663. 34741520 10.1093/nutrit/nuab084 PMC9086769

[CIT0069] Prade T , Muneer F, Berndtsson E, Nynäs AL, Svensson SE, Newson WR, Johansson E. 2021. Protein fractionation of broccoli (*Brassica oleracea*, var. Italica) and kale (*Brassica oleracea*, var. Sabellica) residual leaves—a pre-feasibility assessment and evaluation of fraction phenol and fibre content. Food and Bioproducts Processing 130, 229–243.

[CIT0070] Rubisco Foods. 2020. Official opening of our plant-based protein production facility https://rubiscofoods.com/official-opening-of-our-plant-based-protein-production-facility/

[CIT0071] Sá AGA , Moreno YMF, Carciofi BAM. 2020. Food processing for the improvement of plant proteins digestibility. Critical Reviews in Food Science and Nutrition 60, 3367–3386. 31760758 10.1080/10408398.2019.1688249

[CIT0072] Salvucci ME , Portis AR Jr, Orgen WL. 1986. Light and CO2 response of ribulose-1,5-bisphosphate carboxylase/oxygenase activation in Arabidopsis leaves. Plant Physiology 80, 655–659. 16664680 10.1104/pp.80.3.655 PMC1075178

[CIT0073] Samtiya M , Aluko RE, Dhewa T. 2020. Plant food anti-nutritional factors and their reduction strategies: an overview. Food Production, Processing and Nutrition 2, 1–14.

[CIT0074] Sedlar T , Čakarević J, Tomić J, Popović L. 2021. Vegetable by-products as new sources of functional proteins. Plant Foods for Human Nutrition 76, 31–36. 33245466 10.1007/s11130-020-00870-8

[CIT0075] Serafini-Fracassini D , Del Duca S. 2008. Transglutaminases: widespread cross-linking enzymes in plants. Annals of Botany 102, 145–152. 18492735 10.1093/aob/mcn075 PMC2712369

[CIT0076] Sowersby T , Edmonds R, Huffman L, Fletcher K. 2021. Leaf protein from pasture. Food New Zealand 21, 52–56.

[CIT0077] Stuff. 2021. Pavlova made from grass, and other foods of the future https://www.stuff.co.nz/business/farming/agribusiness/124054241/pavlova-made-from-grass-and-other-foods-of-the-future

[CIT0078] Sun CZ , Wu WJ, Min T, Liu Y, Zhu JH, Lai FR, Wu H. 2015. Functional properties of mulberry (*Morus atropurpurea* Roxb.) leaf proteins extracted by different methods. Modern Food Science and Technology 31, 235–241.

[CIT0079] Tamayo Tenorio A , Gieteling J, De Jong GAH, Boom RM, Van Der Goot AJ. 2016. Recovery of protein from green leaves: overview of crucial steps for utilisation. Food Chemistry 203, 402–408. 26948631 10.1016/j.foodchem.2016.02.092

[CIT0080] Tamayo Tenorio A , Schreuders FKG, Zisopoulos FK, Boom RM, van der Goot AJ. 2017. Processing concepts for the use of green leaves as raw materials for the food industry. Journal of Cleaner Production 164, 736–748.

[CIT0081] Tan Y , Lee PW, Martens TD, McClements DJ. 2022. Comparison of emulsifying properties of plant and animal proteins in oil-in-water emulsions: whey, soy, and RuBisCo proteins. Food Biophysics 17, 409–421.

[CIT0082] Taylor SL , Marsh JT, Koppelman SJ, Kabourek JL, Johnson PE, Baumert JL. 2021. A perspective on pea allergy and pea allergens. Trends in Food Science & Technology 116, 186–198.

[CIT0083] Tome D. 2013. Digestibility issues of vegetable versus animal proteins: protein and amino acid requirements—functional aspects. Food and Nutrition Bulletin 34, 272–274. 23964409 10.1177/156482651303400225

[CIT0084] Trindler C , Annika Kopf-Bolanz K, Denkel C. 2022. Aroma of peas, its constituents and reduction strategies—effects from breeding to processing. Food Chemistry 376, 131892. 10.1016/j.foodchem.2021.131892 34971885

[CIT0085] Udenigwe CC , Okolie CL, Qian H, Ohanenye IC, Agyei D, Aluko RE. 2017. Ribulose-1,5-bisphosphate carboxylase as a sustainable and promising plant source of bioactive peptides for food applications. Trends in Food Science and Technology 69, 74–82.

[CIT0086] Ufaz S , Galili G. 2008. Improving the content of essential amino acids in crop plants: goals and opportunities. Plant Physiology 147, 954–961. 18612072 10.1104/pp.108.118091 PMC2442549

[CIT0087] Vegconomist. 2021. San Diego’s Plantible Secures $21.5M For Alt Meat & Dairy Created From One of World’s Most Sustainable Plant Proteins https://vegconomist.com/investments-finance/san-diegos-plantible-secures-21-5m-for-alt-meat-dairy-created-from-one-of-worlds-most-sustainable-plant-proteins/?utm_source=relatedposts&utm_medium=relatedpostswidget&utm_campaign=crp

[CIT0088] Vinnova. 2021. PlantProteinFactory Step 2 https://www.vinnova.se/en/p/plantproteinfactory-step-2/

[CIT0089] Wang C , Sun C, Lu W, Gul K, Mata A, Fang Y. 2020. Emulsion structure design for improving the oxidative stability of polyunsaturated fatty acids. Comprehensive Reviews in Food Science and Food Safety 19, 2955–2971. 33337053 10.1111/1541-4337.12621

[CIT0090] Wang Q , Xiong YL. 2019. Processing, nutrition, and functionality of hempseed protein: a review. Comprehensive Reviews in Food Science and Food Safety 18, 936–952. 33336999 10.1111/1541-4337.12450

[CIT0091] Wildman SG. 1992. Early events along the trail leading to identification of Rubisco. Protein Science 1, 303–304. 1304911 10.1002/pro.5560010212 PMC2142199

[CIT0092] Wildman SG. 2002. Along the trail from Fraction I protein to Rubisco (ribulose bisphosphate carboxylase-oxygenase). Photosynthesis Research 73, 243–250. 16245127 10.1023/A:1020467601966

[CIT0093] Wu G , Cross HR, Gehring KB, Savell JW, Arnold AN, McNeill SH. 2016. Composition of free and peptide-bound amino acids in beef chuck, loin, and round cuts. Journal of Animal Science 94, 2603–2613. 27285936 10.2527/jas.2016-0478

[CIT0094] Xu Y , Li Y, Bao T, Zheng X, Chen W, Wang J. 2017. A recyclable protein resource derived from cauliflower by-products: Potential biological activities of protein hydrolysates. Food Chemistry 221, 114–122. 27979071 10.1016/j.foodchem.2016.10.053

[CIT0095] Yakhlef M , Giangrieco I, Ciardiello MA, Fiume I, Mari A, Souiki L, Pocsfalvi G. 2021. Potential allergenicity of *Medicago sativa* investigated by a combined IgE-binding inhibition, proteomics and in silico approach. Journal of the Science of Food and Agriculture 101, 1182–1192. 32790067 10.1002/jsfa.10730

[CIT0096] Yang S , Kawamura Y, Yoshikawa M. 2003. Effect of rubiscolin, a delta opioid peptide derived from Rubisco, on memory consolidation. Peptides 24, 325–328. 12668220 10.1016/s0196-9781(03)00044-5

[CIT0097] Yang Y , Marczak ED, Usui H, Kawamura Y, Yoshikawa M. 2004. Antihypertensive properties of spinach leaf protein digests. Journal of Agricultural and Food Chemistry 52, 2223–2225. 15080624 10.1021/jf034551v

[CIT0098] Yeoh H-H , Wee Y-C. 1994. Leaf protein contents and nitrogen-to-protein conversion factors for 90 plant species Food Chemistry 49, 245–250.

[CIT0099] Zayas JF. 1997. Functionality of proteins in food. Berlin: Springer.

[CIT0100] Zhang W , Ding L, Grimi N, Jaffrin MY, Tang B. 2017. A rotating disk ultrafiltration process for recycling alfalfa wastewater. Separation and Purification Technology 188, 476–484.

[CIT0101] Zhang W , Grimi N, Jaffrin MY, Ding L. 2015. Leaf protein concentration of alfalfa juice by membrane technology. Journal of Membrane Science 489, 183–193.

[CIT0102] Zhong M , Wang Y, Hou K, Shu S, Sun J, Guo S. 2019. TGase positively regulates photosynthesis via activation of Calvin cycle enzymes in tomato. Horticulture Research 6. 10.1038/s41438-019-0173-z PMC6804539 31645950

